# Association of social determinants of health and physical functioning among breast cancer survivors

**DOI:** 10.1007/s00520-026-10969-4

**Published:** 2026-07-03

**Authors:** Diane Von Ah, Claire Han, Leorey Saligan, Ashley Pariser Davenport, Nicole Williams, Michelle Naughton, Susan Storey, Yesol Yang, Macy Corley, Jesse J. Plascak

**Affiliations:** 1 College of Nursing, The Ohio State University, 394 Newton Hall, 1585 Neil Avenue, Columbus, OH 43210 USA; 2 School of Nursing, Rutgers University, 180 University Avenue, Newark, NJ 07102 USA; 3 Division of Medical Oncology, James Comprehensive Cancer Center, The Ohio State University, Columbus, OH 43210 USA; 4 Department of Internal Medicine, College of Medicine, The Ohio State University, 3650 Olentangy River Rd, Suite 200, Columbus, OH 43214 USA; 5 School of Nursing, Indiana University, Indianapolis, IN 46202 USA; 6 College of Medicine, The Ohio State University, 3650 Olentangy River Rd, Suite 200, Columbus, OH 43214 USA

**Keywords:** Social determinants of health, Education, Income, Physical functioning, Breast cancer survivors

## Abstract

**Purpose:**

The purpose of this study was to address the relationship between social determinants of health (both individual and neighborhood levels) to understand more fully how social conditions are associated with physical functioning among breast cancer survivors (BCS).

**Methods:**

We conducted a secondary analysis of a national sample of breast cancer survivors recruited via Institutional Review Board–approved social media (i.e., Facebook) and online cancer-affiliated resource sites (e.g., Pink Ribbon Connection, Dr. Susan Love Foundation). BCS provided their address and demographic factors and completed the physical functioning survey (PF-10). Neighborhood social determinants of health (SDOH) included Yost National Rank Index and neighborhood factors (walkability and rural vs. urban status) using Geocoding. Descriptive statistics and linear regression models were used.

**Results:**

BCS who were older (*p* = 0.038) and single (*p* = 0.008) had poor physical functioning. Socioeconomic factors including higher education (*p* = 0.001), higher income (*p* = 0.034), and higher neighborhood socioeconomic status (Yost Index, *p* = 0.016) were associated with greater physical functioning. Neighborhood factors (walkability and rural vs. urban) were not associated with physical functioning in BCS.

**Conclusion:**

Physical functioning of BCS was linked to age, social network (marital status), and socioeconomic status. Future interventional research is needed that accounts for individual and socioeconomic factors to mitigate their effects on the physical functioning of BCS.

## Introduction

Breast cancer survivors (BCS) represent the largest population of cancer survivors worldwide, with over 4 million living in the USA alone [1]. Due to advances in early detection and treatment, BCS are living longer, often with physical function impairments that last well into cancer survivorship [2, 3].

Physical functioning has been shown to be central to maintaining independence [4] and decreased physical functioning has been associated with increased frailty, disability, and poorer quality of life in cancer survivors [5–7]. Researchers have long identified that BCS often experience declines in physical function, that may affect both emotional and physical well-being [8]. In fact, Cao and colleagues (2024) analyzed data from 47,768 cancer survivors and 2,432,754 non-cancer adults age 18 years and older from the 2017 to 2022 Behavioral Risk Factor Surveillance System and found that over a quarter of the cancer survivors had physical function impairments including mobility disability (27.9% vs. 13.4%) and self-care disability (7.4% vs. 3.8%), which were both higher among cancer survivors compared with non-cancer adults [9]. Thus, understanding factors that impact physical functioning is imperative to improving the quality of life of BCS.

Social determinants of health (SDOH) are among the key factors involved in the pathogenesis, diagnosis, and outcomes from treatment for cancer [10]. Recently, there has been more emphasis to explore the impact of social determinants for survivorship care [11]. SDOH, as defined by the Centers for Disease Control and Prevention (CDC), refer to the conditions in the environment that may influence a wide range of health, functioning, and quality of life outcomes and risks [12]. SDOH can be measured at both individual and area levels, capturing distinct but complementary influences on health [12]. Individual-level data encompass age, race/ethnicity, socioeconomic status, education, income, and employment, providing detailed survivor-specific insights [10]. Area-level SDOH, derived from geographic units such as census tracts, capture neighborhood socioeconomic composition and built environment characteristics, walkability, as well as rurality, offering more objective measures that can be incorporated into healthcare settings to identify disparities and tailor interventions [11]. Both levels of socioeconomic factors have demonstrated significant associations with health outcomes among cancer survivors, underscoring the importance of addressing multi-level social contexts to improve survivorship care and reduce health inequities.

Notably, SDOH that may impact physical functioning of BCS may include social and community relationships and context, economic stability, education access and quality, and neighborhood and built environment. Social relationships have been linked to cancer outcomes including disease progression, mortality [13], and more recently to functional impairments in BCS [14]. Guida et al. (2019) analyzed data from 1481 community-dwelling older adults (*n* = 201 cancer survivors) and noted that declines in frequent social contact were associated with higher odds of functional impairment among cancer survivors (OR = 1.92, 95% CI = 1.15–3.20), suggesting that social relationships may play an important role in reducing functional impairments in BCS. Economic stability, which includes the individual or family income, education, and employment as well as the economic conditions within a community, may have a direct impact on the health and overall welfare of its residents [15]. Researchers have identified that socioeconomic indices increase the overall variation in the incidence and mortality of breast cancer [16], but few studies have fully explored these factors in the context of cancer survivorship. In addition, the neighborhood-built environment including walkability and rural vs. urban environments may influence physical activity and physical functioning of survivors. Research has shown that neighborhoods that offer more access and pedestrian connectivity to recreational centers and parks and provide more personal and traffic safety promote physical activity and ultimately may impact physical functioning and overall health of survivors [17–19]. In addition, rural vs. urban disparities which have been well documented with the diagnosis and treatment of cancer may also impact the health of long-term cancer survivors [20, 21]. Researchers have emphasized the need to fully examine the impact of social determinants of health on survivorship outcomes such as physical functioning [11, 21].

Therefore, the purpose of this study was to address the relationship between SDOH and physical functioning to understand more fully how social conditions are associated with physical functioning among BCS. Understanding social factors that contribute to physical functioning is imperative to both identifying those at risk as well as developing clinically meaningful intervention strategies to support BCS.

## Methods

This study was a cross-sectional, secondary data analysis that is part of a parent study examining factors associated with cancer-related cognitive impairment in cancer survivors (ClinicalTrials.gov Identifier: NCT04611620; registration date: 2021.07.27). The recruitment methods and eligibility criteria of the parent study are described in our previous paper [13]. Briefly, we enrolled breast and colorectal cancer survivors who were ≥ 21 years old, were at ≥ 6 months after completing adjuvant/neo-adjuvant therapies (current Aromatase Inhibitors or Tamoxifen use at the time of enrollment of BCS was allowed), and had self-reported cancer-related cognitive impairment. In this analysis, only Stage I–III BCS were included. Prior to study entry, each participant completed an online informed consent obtained through the secure Health Insurance Portability and Accountability Act-compliant Research Electronic Data Capture (REDCap®) system. Once consent was obtained participants completed survey questionnaires that were delivered electronically, completed an online cognitive assessment, and provided a buccal swab for genetic analysis. For this analysis, only the survey data was analyzed. The study was approved by a large comprehensive cancer center in the Midwest and the University Institutional Review Board.

### Study sample

Among the 555 BCS who consented and completed the survey data, 518 (93.2%) provided an address that was geocoded to the block group and census tract levels from the 2010 and 2020 US Census TIGER files [14] and were included in this final analysis. The surveys in this analysis included demographic information and characteristics as well as a measure of physical functioning.

### Measures

#### Social determinants of health

SDOH were measured both at the individual level and at the neighborhood level. Individual sociodemographic data such as age, sex, race, marital status, highest level of education completed, current work status, and income were gathered through self-report. Household income was originally reported in categorical ranges (< $15,000, $15,001–$30,000, $150,001–200,000, and > $200,000) but converted to continuous values based on the midpoint of the range for this study.

Neighborhood-level socioeconomic status was also measured by census tract-level Yost Index [15]. The Yost Index has been shown to have high face validity, capturing the combined impact of average educational level, median household income, poverty rate, median housing value, median rent, unemployment rate, and occupational prestige [16]. Yost measures were calculated annually, nationally ranked, and linked to participants based on residential address at study consent (2020 or 2021) [15]. We extended previous measures and methods to yield country-wide Yost estimates for 2018 and 2019 using the American Community Survey 5-year estimates (2016–2020 [2018] and 2017–2021 [2019]) [15]. Variables to calculate Yost values were gathered from the National Historical Geographic Information System (NHGIS) and the U.S. Census Bureau websites and included percent working class, percent aged 16 years who are unemployed, percent of persons below 150% of poverty line, median household income, education index (weighted school years), median house value, and median rent [17–19]. Factor analysis of all variables by year, each centile rank-transformed prior to analysis, resulted in a single factor (analyses were limited to US states). Yost indices were centile ranked nationally with 1= lowest socioeconomic composition and 100 = highest socioeconomic composition, and analyzed as nationally based quartiles. Year-specific Yost linkages were 2-year lagged; 2021 diagnosis dates were assigned 2019 Yost.

Two additional neighborhood-level variables were assessed: walkability and rural/urban status. Walkability was from the US Environmental Protection Agency National Walkability Index provided at the block group level [20]. This index is a macroscale walkability measure calculated from street intersection density, proximity to transit stops, and diversity of land uses [21]. Rural-urban was estimated with census tract linkages to the 2010 Rural-urban commuting area codes (RUCA), with RUCA value of 1/“Metropolitan area core” indicating the most urban, and RUCA 2–99 indicating the non-urban [22].

#### Physical functioning

Physical functioning was measured by the Physical Functioning-10 (PF-10) Questionnaire. The PF-10 is a subscale of the Medical Outcomes Study 36-item Short Form Health Survey (SF-36). The SF-36, and its subscales including the PF-10, has been established as a comprehensive measure of general health that has shown reliability and validity in various populations, including cancer patients [23]. The PF-10 measures the participants’ perceived limitations of physical functioning during the past 4 weeks with a 3-point scale (yes, limited a lot; yes, limited a little; and no, not limited at all), using the original 0–100 scores, with higher scores indicating less limitation or disability or greater functioning. Cronbach’s alpha was 0.93 in this study.

### Data analysis

The study sample was summarized by levels of covariates (mean, percent, standard deviation, sample size). As both the Yost and walkability measures are from national datasets, we compared sample characteristics to US-wide values. A linear regression model of physical functioning was built to test associations with individual and neighborhood-level SDOH factors and adjust for potential confounders. Beta and 95% confidence intervals (CI) were calculated. Analyses did not account for clustering within census tracts as only 10 census tracts had 2 or 3 participants, while the remainder of census tracts each had 1 participant.

Missingness among the 518 was as follows: 62 (12.0%) household income, 44 (8.5%) Yost index, 27 (5.2%) physical function index, 12 (2.3%) race-ethnicity, and 8 (1.5%) marital status. Multiple imputation using chained equations was performed to generate 25 imputed datasets. Regression coefficients of each imputed dataset were combined using Rubin’s Rule. Conditional normality of functional limitations was examined through residual plots and transformations considered. SAS 9.4 and ArcGIS 10.6 were used for data management and analyses.

## Results

A total of 518 Stage I–III BCS were included in the analysis. Table 1 presents the sample characteristics including sociodemographic, contextual, and physical functioning data. In summary, most participants self-identified as non-Hispanic, White (89.2%), married or living with a partner (71.0%), and resided in less urban areas (70.5%). Approximately 33% reported obtaining a master’s degree or higher education and an average annual household income of $88,655. Participants tended to reside in slightly less walkable areas compared to the US population; with 7.5% residing in the most walkable block groups vs. 10.7% of the US population and 25.7% resided in the least walkable block groups vs. 23.0% of the US population [20]. The average participant’s median Yost national centile was 67%, indicating the residence was in a higher census tract socioeconomic composition compared to the US population overall (50.0%).

**Table 1:** Sample characteristics by sociodemographic, contextual, and physical functioning, *n* = 518^1^

Variable	*N* (%)
Age, y (mean, SD^2^)	55.8 (9.9)
Race-ethnicity	
Non-Hispanic, White	462 (89.2)
Hispanic, Black/African American, Asian, American India, Alaskan Native, Native Hawaiian/Other Pacific Islander, More than one race	44 (8.5)
Marital status	
Married, living with partner	368 (71.0)
Single, divorced, widowed, other	142 (27.4)
Education	
Up to some college or associate degree	181 (34.9)
4-year college graduate or bachelor degree	165 (31.8)
Master degree or more	172 (33.2)
Household income, $ (mean, SD^2^)	88,655 (55,868)
Yost National Ranking Quartiles	
1–25 (lowest)	54 (10.4)
25–50	97 (18.7)
51–75	142 (27.4)
76–100 (highest)	181 (34.9)
Walking Index	
Least walkable	133 (25.7)
Below average walkable	232 (44.8)
Above average walkable	114 (22.0)
Most walkable	39 (7.5)
Rural urban commuting area	
Urban (RUCA = 1.0)	153 (29.5)
Less urban (RUCA > 1.0)	365 (70.5)
Physical functioning PF-10 (mean, SD^2^)	68.0 (27.0)

^1^Frequencies may not sum to 518 due to missingness

^2^SD is standard deviation

Table 2 presents the regression analysis of the association of SDOH and physical functioning in BCS, mutually adjusted for factors listed. BCS who were older (*β* = −2.46, CI = −4.74 to 0.17, *p* = 0.035) and single (*β* = −7.28; CI = −12.62 to 1.94, *p* = 0.008) had poor physical functioning. In addition, socioeconomic factors including lower education (*p* = 0.001) and lower income (*p* = 0.034) were associated with poor physical functioning. Socioeconomic status as measured by the Yost Index was not significant using the categorical variable obtained by cut-off scores; however, the linear regression model using continuous Yost values (e.g., 1–100) suggested that increases in socioeconomic status was associated with greater physical functioning; *β* = 0.12 (95% CI = 0.02–0.23), *p* < 0.016). Other neighborhood factors of walkability and rural vs. urban were not associated with physical functioning in BCS.

**Table 2:** Association of social determinants of health and physical functioning in breast cancer survivors, *n* = 518^1^

Variable	*β* (95% CI)	*p*-value
Individual-level SDOH		
Age, per 10 years	−2.46 (−4.74 to 0.17)	**.035**
Race-ethnicity		
Non-Hispanic, White	Reference	
Hispanic, Black/African American, Asian, American Indian, Alaskan Native, Native Hawaiian/Other Pacific Islander, More than one race	1.74 (−6.27 to 9.76)	0.670
Marital status		
Married, living with partner	Reference	
Single, divorced, widowed, other	−7.28 (−12.62 to 1.94)	**.008**
Education		
Up to some college or associate degree	Reference	
4-year college graduate or bachelor degree	9.97 (4.27–15.66)	**.001**
Master degree or more	11.63 (5.66–17.59)	** <.001**
Household income, per $10,000	0.55 (0.04–1.06)	**.034**
Neighborhood-level SDOH		
Yost National Ranking Quartiles^2^		
1–25 (lowest)	Reference	
26–50	2.93 (−5.93 to 11.78)	.517
51–75	6.97 (−1.40 to 15.34)	.103
76–100 (highest)	8.36 (−0.36 to 17.09)	**.060**
Walking Index		
Least walkable	Reference	
Below average walkable	3.37 (−2.34 to 9.09)	.248
Above average walkable	5.73 (−1.30 to 12.76)	.110
Most walkable	−1.54 (−11.08 to 8.00)	.752
Rural urban commuting area		
Urban (RUCA = 1.0)	Reference	
Rural (RUCA > 1.0)	−1.14 (−6.92 to 4.62)	.697

^1^From linear regressions of 25 imputed datasets

^2^Model with Yost as continuous values: *β* = 0.12 (95% CI = 0.02–0.23), *p* =.016

## Discussion

SDOH have been shown to be important factors that impact cancer outcomes. However, much of the existing focus has centered on disparities in cancer prevention, diagnosis, and treatment. Recent calls have emphasized the need for more research regarding SDOH and cancer survivorship outcomes [24]. This study was designed to examine the linkages between SDOH and physical functioning. Physical functioning is essential for maintaining independence and the ability to perform everyday activities. Declines in physical functioning have been linked to greater frailty, increased disability, reduced participation in daily life, and poorer quality of life. Understanding the factors that influence physical functioning is therefore critical to identifying survivors at risk and developing strategies to improve overall health, independence, and well-being in BCS. The findings of this study revealed significant associations between age, social support (marital status), education, income, and socioeconomic status with physical functioning, whereas neighborhood factors such as walkability and rurality were not linked with physical function in BCS.

In this study, socioeconomic factors including education, income, and the Yost Index which captured the core dimensions of education, income, and employment were all directly associated with physical functioning in BCS. Disparities in cancer survival by socioeconomic status, education, and poverty have been well established [25]. Yet, only a few studies have examined the impact on physical function and quality of life of cancer survivors. In a large US cohort study, Cao and colleagues identified that physical function limitations (higher prevalence of mobility and self-care disabilities) were observed in cancer survivors who had lower levels of education and income, among other treatment-related factors [9]. Similarly, in a large Danish cohort study of 2235 BCS, those from a lower social class (shorter education) had an increased likelihood (OR = 1.7) for reporting poor physical function [26]. Importantly, disparities of education, income, and employment have also been shown to be associated with inadequate health insurance, lack of primary physician, and access to health care, all of which may continue to impact the health and well-being of cancer patients well into survivorship [24]. In addition, cancer treatment demands can create an enormous financial burden with both direct costs of care (e.g., deductibles, copays, and out of insurance plan coverage) and indirect costs (e.g., transportation, child care, and time away from work) [27], further compounding existing socioeconomic disparities and heightening their potential impact on health and physical functioning of long-term BCS. In addition, we noted that higher education was significantly associated with better physical functioning. This finding is consistent with a previous study by Levinsen and colleagues who demonstrated that cancer survivors with less education were more likely to report impaired physical function [26]. In fact, up to 72% of the cancer survivors (*n* = 27,857) with lower educational levels (≤ 9 years) reported impaired physical functioning. These authors and others have proposed that educational disparities may be an underlying factor contributing to lower health literacy and poorer self-assessed health in long- term cancer survivors [26, 28]. Thus, survivorship care planning must include an assessment of the survivors reading level and overall health literacy as well as uncovering the overall socioeconomic status and resources available to survivors to improve physical function and overall health. Promoting physical activity alongside treatment is essential for improving long-term survival, physical function, and health-related quality of life in cancer survivors. Achieving this requires individualized, patient-centered care that addresses personal and societal factors affecting quality of life [24, 29].

Age also played a role in physical functioning after breast cancer treatment. As anticipated, we noted that older BCS reported poorer physical functioning. It is well understood that older cancer survivors are often plagued with the effects of aging which are then compounded with potentially debilitating treatments and side-effects (e.g., pain, neuropathy, fatigue) that may further impact functional ability [2]. Similarly, in a large cohort study (total *n* = 7495 with 829 cancer survivors), Gell and colleagues noted that compared to non-cancer controls, older cancer survivors reported worse physical capacity and poorer performance on a battery of physical function tests, and that women survivors were disproportionately affected [30]. Taken together, older cancer survivors, especially female survivors, may be more vulnerable to physical function impairments and thus more interventions designed to maintain functional ability, reduce comorbidities, and optimize independence are needed [31].

Social relationships have been shown to impact health and well-being of cancer survivors [24]. In this study, we noted that those BCS who were married also reported better physical function. Similar findings regarding the protective effects of social support have been noted in previous studies [32, 33]. Guida and colleagues (2019) identified that adding new relationships was protective of disability in both cancer and non-cancer older adults and that declines in the frequency of contact were associated with having at least one functional impairment among cancer survivors [33]. Social isolation, on the other hand, has been associated with poorer health-related outcomes [34, 35]. Several studies have also documented that cancer survivors with a strong social network (being married) have improved survival [36, 37]. Hurtado-de-Mendoza and colleagues (2023) identified that emotional/informational and tangible support were associated with dimensions of health-related quality of life including functional well-being in 545 BCS [32]. Future research with a more nuanced approach to examining the social network and various types of social support and their role in cancer survivorship is needed to inform intervention research in cancer survivorship [38].

Neighborhood factors such as walkability and rurality were not associated with physical functioning in this study of BCS. Several factors may explain these findings. Physical functioning in cancer survivors is often influenced by treatment-related effects—such as fatigue, neuropathy, or musculoskeletal limitations. The impact of these symptoms may have potentially overshadowed neighborhood influences [38]. In addition, regardless of environmental supports, survivors may engage in physical activity through indoor or structured programs, rely on motorized transportation, or benefit from strong social support, reducing reliance on the immediate neighborhood for activity[39]. Furthermore, the relationship between rural vs. urban cancer disparities is complex. The American Society of Clinical Oncology (ASCO) in 2018 developed a model to address factors contributing to rural cancer care disparities to support future research in this area [40]. Additional neighborhood characteristics may have been more salient for this population. For example, Namin and colleagues conducted a review of the literature and found that those studies utilizing neighborhood socioeconomic status SES noted a relationship between neighborhood deprivation and the long-term effects of treatments and quality of life and physical functioning after cancer survival [11]. For example, Pruitt et al. found that BCS living in neighborhoods with higher poverty were more likely to report lower physical functioning 2 years later (*β* =  −0.19, *p* < 0.001); however, this effect was fully mediated by physical activity and BMI [41]. More research is needed to understand the relationship between neighborhood-built environments and physical functioning post-cancer treatment.

## Strengths and limitations

The strengths of this study include the large national sample of BCS using multiple variables assessing SDOH. In addition, this study focused on physical functioning, an important indicator of health for cancer survivors. However, the study was limited by the mostly White and female sample with early-stage breast cancer and thus does not represent all BCS. In addition, the cross-sectional design limits interpretation to associations between variables versus establishing a causal effect. Future research should also assess the number and type of comorbidities when examining physical functioning. Lastly, research on the effects of neighborhoods on health and well-being remains challenging due to persistent methodological limitations [42]. Neighborhoods are often defined by using arbitrary spatial boundaries, but the appropriate spatial scale for measuring neighborhood exposure is complex. Individuals’ interactions with their living and working environments are difficult to delineate [42], further complicating the assessment of neighborhood influence. Future longitudinal studies with more diverse participants will be vital in exploring the role of SDOH and their relationship to physical function and quality of life across the cancer care trajectory. In addition, future studies should consider expanded assessment of social network and support indices as well as objective measures of physical functioning to determine if these associations remain.

## Conclusion

This study demonstrates that physical functioning in BCS is significantly influenced by individual factors such as age and marital status, as well as socioeconomic status including education, income, and neighborhood-level socioeconomic advantage measured by the Yost Index. Contrary to expectations, neighborhood walkability and rural vs. urban residence were not associated with physical functioning in this population. These findings underscore the importance of addressing both individual (age, social support factors) and socioeconomic (both individual and neighborhood levels) SDOH to support the physical health of BCS. Recognizing these influences is critical for survivorship care, suggesting that interventions addressing educational, financial, and social needs could help maintain or improve physical functioning. This study highlights the importance of incorporating socioeconomic assessments into survivorship plans and calls for further research to develop targeted strategies to support survivors’ long-term functional health.

## Data Availability

The datasets during and/or analyzed during the current study are available from the corresponding author on reasonable request.
